# 3-(Amino­carbon­yl)pyridinium diaqua-bis­(pyridine-2,6-dicarboxyl­ato)bis­muthate(III) monohydrate

**DOI:** 10.1107/S160053681202630X

**Published:** 2012-06-20

**Authors:** Janet Soleimannejad, Samira Gholizadeh

**Affiliations:** a School of Chemistry, College of Science, University of Tehran, Tehran, Iran

## Abstract

The asymmetric unit of the ionic title compound, (C_6_H_7_N_2_O)[Bi(C_7_H_3_NO_4_)_2_(H_2_O)_2_]·H_2_O or (acpyH)[Bi(pydc)_2_(H_2_O)_2_]·H_2_O, contains an [Bi(pydc)_2_(H_2_O)_2_]^−^ anion (where pydcH_2_ is pyridine-2,6-dicarb­oxy­lic acid), a protonated 3-(amino­carbon­yl)pyridine as counter-ion, (acpyH)^+^, and one uncoordinated water mol­ecule. The anion is an eight-coordinate complex with a square-anti­prismatic geometry around the Bi^III^ atom. In the crystal, extensive O—H⋯O and N—H⋯O hydrogen bonds, as well as ion pairing, C=O⋯π inter­actions [O⋯centroid distance = 3.583 (5) Å], π–π stacking [centroid–centroid distance = 3.864 (3) Å], and C—H⋯π and C—H⋯O inter­actions, play an important role in the formation and stabilization of the three-dimensional supra­molecular structure.

## Related literature

For related structures, see: Aghabozorg, Ramezanipour *et al.* (2008 ▶); Aghabozorg, Nemati *et al.* (2008 ▶); Ranjbar *et al.* (2003 ▶); Sharif *et al.* (2007 ▶); Sheshmani *et al.* (2005 ▶). For graph-set motifs, see: Bernstein *et al.* (1995 ▶). 
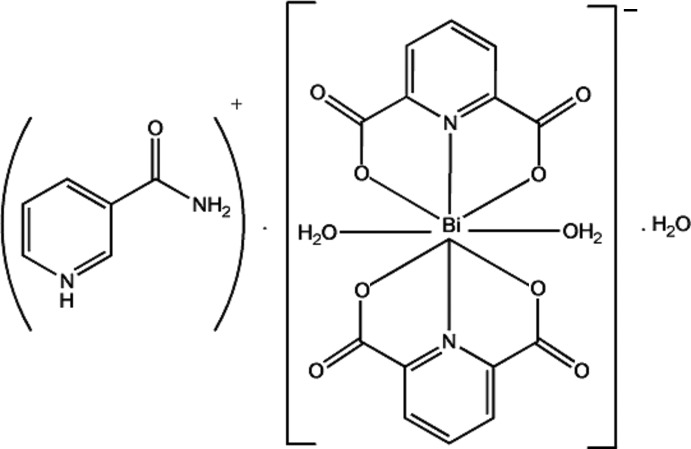


## Experimental

### 

#### Crystal data

(C_6_H_7_N_2_O)[Bi(C_7_H_3_NO_4_)_2_(H_2_O)_2_]·H_2_O
*M* *_r_* = 716.37
Triclinic, 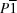

*a* = 8.7702 (6) Å
*b* = 10.7954 (7) Å
*c* = 11.9203 (8) Å
α = 80.409 (3)°
β = 80.952 (3)°
γ = 81.730 (3)°
*V* = 1090.92 (13) Å^3^
*Z* = 2
Mo *K*α radiation
μ = 8.16 mm^−1^
*T* = 296 K
0.32 × 0.20 × 0.20 mm

#### Data collection

Bruker SMART CCD area-detector diffractometer
Absorption correction: multi-scan (*SADABS*; Sheldrick, 1996 ▶) *T*_min_ = 0.180, *T*_max_ = 0.292
8311 measured reflections
4994 independent reflections
4689 reflections with *I* > 2σ(*I*)
*R*_int_ = 0.046

#### Refinement

*R*[*F* ^2^ > 2σ(*F* ^2^)] = 0.040
*wR*(*F* ^2^) = 0.104
*S* = 1.05
4994 reflections
335 parameters
H-atom parameters constrained
Δρ_max_ = 3.29 e Å^−3^
Δρ_min_ = −4.30 e Å^−3^

### 

Data collection: *SMART* (Bruker, 1998 ▶); cell refinement: *SAINT* (Bruker, 1998 ▶); data reduction: *SAINT*; program(s) used to solve structure: *SHELXS97* (Sheldrick, 2008 ▶); program(s) used to refine structure: *SHELXL97* (Sheldrick, 2008 ▶); molecular graphics: *SHELXTL* (Sheldrick, 2008 ▶) and *Mercury* (Macrae *et al.*, 2008 ▶); software used to prepare material for publication: *SHELXTL* and *publCIF* (Westrip, 2010 ▶).

## Supplementary Material

Crystal structure: contains datablock(s) I, global. DOI: 10.1107/S160053681202630X/su2401sup1.cif

Structure factors: contains datablock(s) I. DOI: 10.1107/S160053681202630X/su2401Isup2.hkl

Additional supplementary materials:  crystallographic information; 3D view; checkCIF report

## Figures and Tables

**Table 1:** Hydrogen-bond geometry (Å, °) *Cg*1 is the centroid of the N2/C9–C13 ring.

*D*—H⋯*A*	*D*—H	H⋯*A*	*D*⋯*A*	*D*—H⋯*A*
O1*S*—H1*A*⋯O11^i^	0.85	2.14	2.961 (7)	164
O1*S*—H1*B*⋯O8^ii^	0.85	2.13	2.979 (7)	173
N3—H3*C*⋯O3	0.86	1.91	2.766 (7)	173
N4—H4*A*⋯O5^i^	0.86	2.29	3.035 (6)	144
N4—H4*B*⋯O1^iii^	0.86	2.05	2.874 (6)	160
O9—H9*A*⋯O4^iv^	0.85	1.99	2.760 (5)	151
O9—H9*B*⋯O11^iv^	0.85	1.96	2.811 (6)	177
O10—H10*A*⋯O1*S*	0.85	2.05	2.782 (7)	144
O10—H10*B*⋯O8^v^	0.85	2.02	2.860 (6)	172
C5—H5⋯O7^vi^	0.93	2.57	3.170 (7)	122
C11—H11⋯O2^vii^	0.93	2.49	3.159 (7)	129
C35—H35⋯O4	0.93	2.27	3.004 (8)	136
C39—H39⋯O1^iii^	0.93	2.57	3.456 (7)	160
C38—H38⋯*Cg*1^viii^	0.93	2.70	3.549 (7)	153

Symmetry codes: (i) 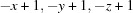; (ii) 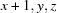; (iii) 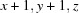; (iv) 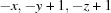; (v) 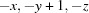; (vi) 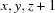; (vii) 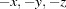; (viii) 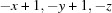.

**Fig. 1.**
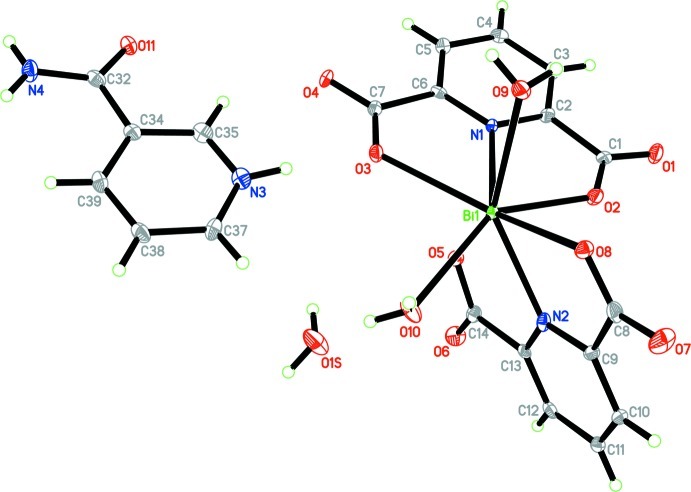
Molecular structure of the title compound with atom numbering. Displacement ellipsoids are drawn at the 50% probability level.

**Fig. 2.**
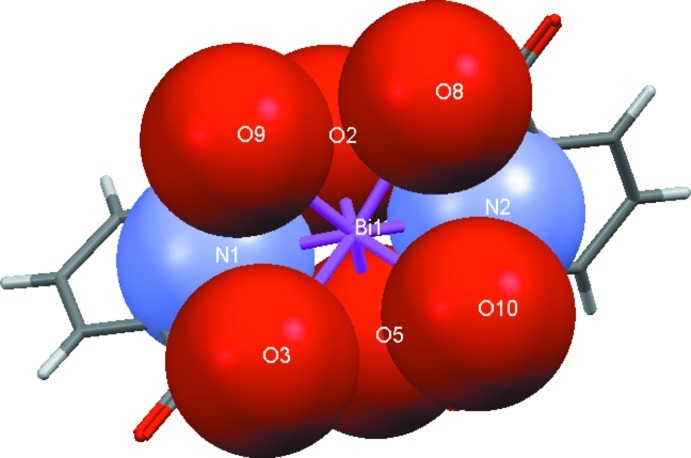
Coordination polyhedron around the bismouth(III) atom, Bi1, illustrating the square antiprism.

**Fig. 3.**
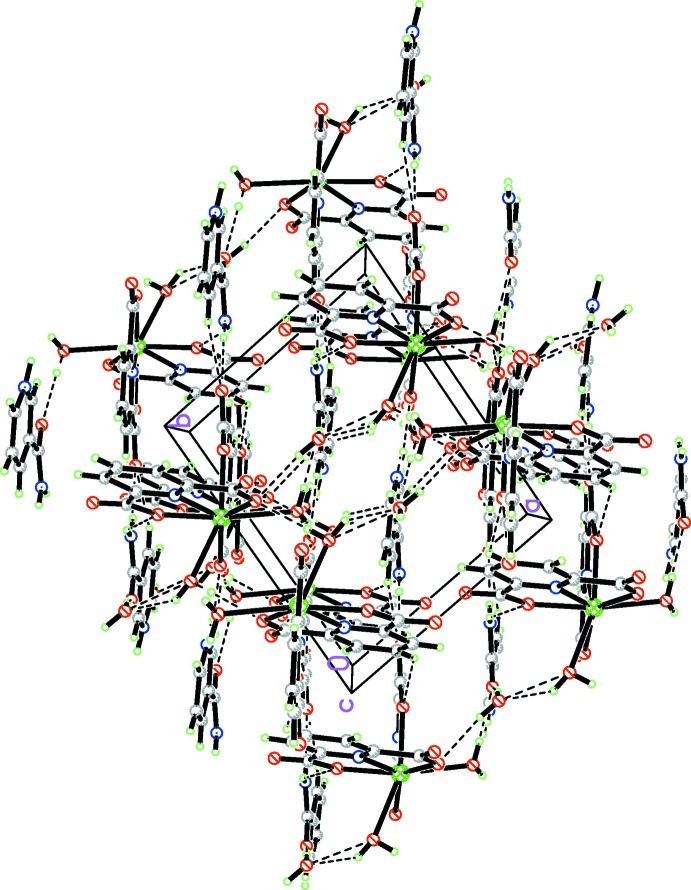
A view along the c axis of the crystal packing of the title complex. Dashed lines indicate O-H···O and N-H···O hydrogen bonds [H atoms not involved in these interactions have been omitted for clarity].

**Fig. 4.**
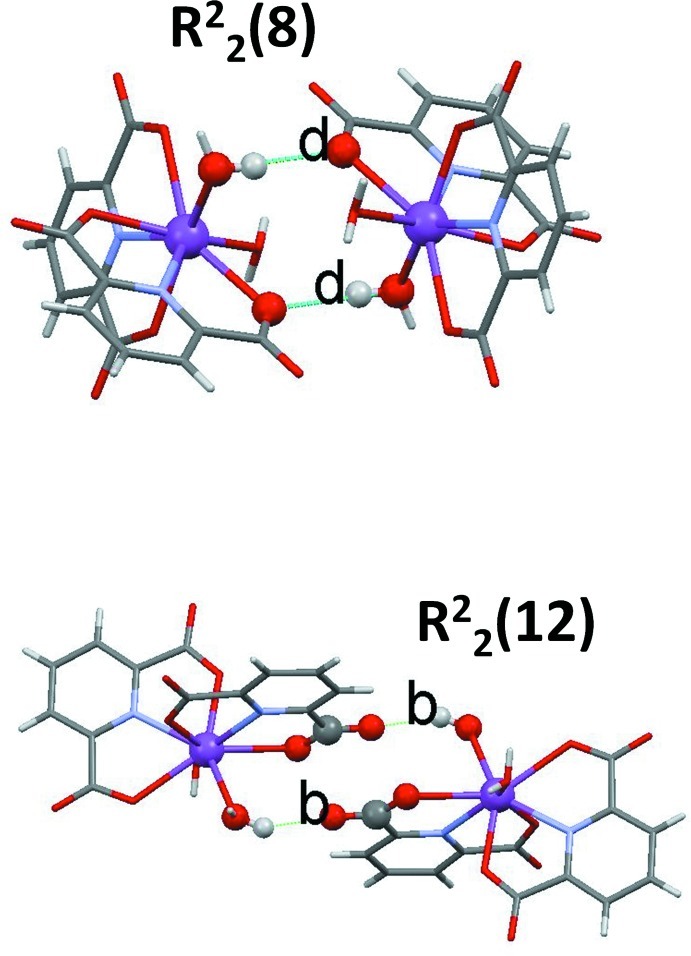
The graph sets motifs formed by intermolecular O—H···O hydrogen bonds involving inversion related anions.

**Fig. 5.**
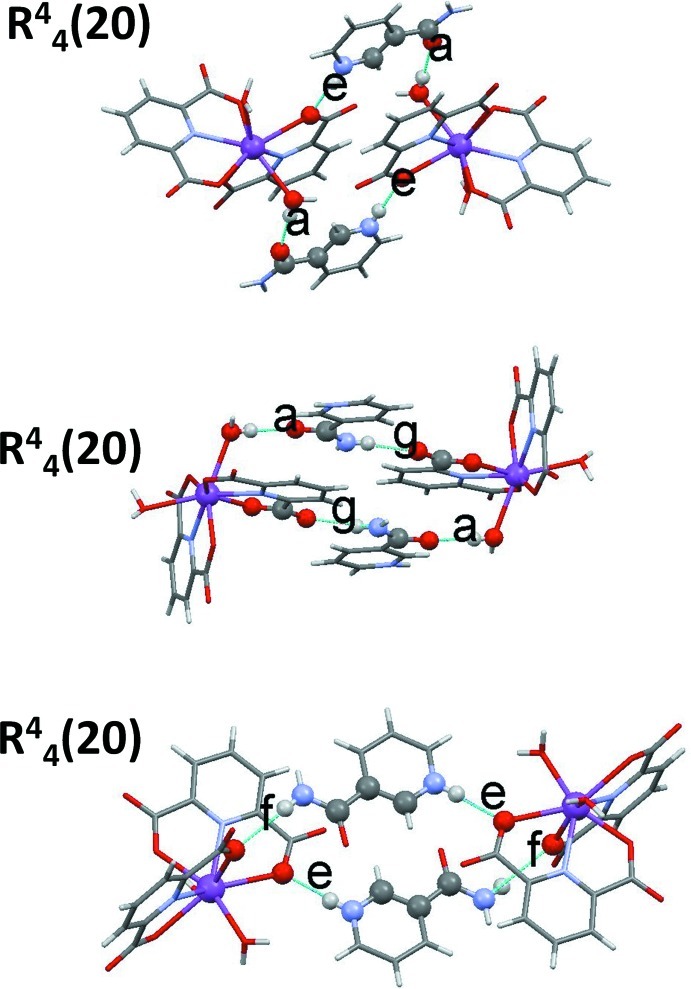
The graph sets motifs formed by intermolecular O—H···O and N—H···O hydrogen bonds involving neighbouring anions and cations.

## supplementary crystallographic information

### Comment

There are a few reports on the coordination of pyridine-2,6-dicarboxylic acid (H_2_pydc), to a Bi^III^ atom, for example (Ranjbar *et al.*, 2003; Sheshmani *et al.*, 2005; Sharif *et al.*, 2007; Aghabozorg, Ramezanipour *et al.*, 2008; Aghabozorg, Nemati *et al.*, 2008; including 2,6-diaminopyridine; 1,10-phenanthroline; 2,4,6-triamino-1,3,5-triazine; 1-methyl-4-oxo-2-imidazolidinimine and piperazine counter ions, respectively). Herein we report on the crystal structure of a similar compound that this time includes a different counter ion, namely 3-(aminocarbonyl)pyridinium.

In the title compound, illustrated in Fig. 1, the asymmetric unit contains a [Bi(pydc)_2_(H_2_O)_2_]^-^ anion, 3-(aminocarbonyl)pyridinium as the counter-ion (acpyH)^+^, and one uncoordinated water molecule. The coordination environment of the Bi^III^ atom may be described as a square antiprism, being composed of two almost parallel planes; atoms O3, O9, O8 and O10 define one plane [mean deviation of 0.257 (4) Å], while atoms N1, O2, N2 and O5 define the other plane [mean deviation of 0.227 (4) Å]. The angle between these two mean planes is 2.07 (18)°, with the Bismuth ion located 1.0102 (2) Å from the first plane and 1.3863 (2) Å from the second. This shows that the Bi^III^ atom is located near the centre of the square antiprism. The twist angle between the two mean planes [O3/Bi1/O8 and N1/Bi1/N2] is 47.36 (1)°, approaching the value of 45° for an ideal square antiprism (Fig. 2).

In the crystal, there are a wide range of non-covalent interactions leading to the formation of a three-dimensional supramolecular structure (Table 1 and Fig. 3). They consist of O—H···O and N—H···O hydrogen bonds and C-H···O and C-H···π interactions (Table 1). There are also C1═O1···π interactions involving the pyridine ring (N1,C2-C6) [distance O1···π being 3.583 (5) Å] and π–π stacking interactions involving inversion related pyridinium rings [N2/C9-C13] with a centroid-centroid distance of 3.864 (3) Å, an interplanar separation of 3.379 (2) Å, and a slippage of 1.875 Å.

In the crystal, the centrosymmetric hydrogen-bonded rings formed by adjacent anions can be described by the basic R^2^_2_(8) graph-set motif (Fig. 4; Bernstein *et al.*, 1995). The carboxylate O atom participates in hydrogen bonding with a neighbouring anion through an O—H···O hydrogen bond. This interaction also links anions into another centrosymmetric hydrogen-bonded ring which can be described by a complex graph-set motif R^2^_2_(12) - see Fig 4. The centrosymmetric hydrogen-bonded rings formed by two adjacent anions and two adjacent cations, including both O—H···O and N—H···O hydrogen bonds, can be described by R^4^_4_(20) ring motifs - see Fig. 5. The aggregation of these ring motifs results in an overall three-dimensional hydrogen-bonded supramolecular structure.

### Experimental

A solution of nicotinamide (70 mg, 0.573 mmol) and pyridine-2,6-dicarboxylic acid (95 mg, 0.573 mmol) in water (6 ml) was heated at 323 K for 1h. BiCl_3_ (180.69 mg, 0.573 mmol) was dissolved in DMSO/water (ratio 1:10, 5 ml) and added to the above solution. The resulting mixture was heated for a further 2 h. It was then filtered off and the filtrate kept at room temperature. Colourless crystals, suitable for X-ray analysis, were obtained after 5 days.

### Refinement

The water and NH H-atoms were located in a difference Fourier map and were refined as riding atoms with distance restrains: O—H = 0.85 (2) Å and N—H = 0.86 (2) Å. The C-bound H-atoms were included in calculated positions and treated as riding atoms: C—H = 0.93 Å, with *U*_iso_(H) = 1.2 *U*_eq_(C).

### Crystal data

**Table:**

(C_6_H_7_N_2_O)[Bi(C_7_H_3_NO_4_)_2_(H_2_O)_2_]·H_2_O	*Z* = 2
*M**_r_* = 716.37	*F*(000) = 692
Triclinic, *P*1	*D*_x_ = 2.181 Mg m^−^^3^
*a* = 8.7702 (6) Å	Mo *K*α radiation, λ = 0.71073 Å
*b* = 10.7954 (7) Å	Cell parameters from 5622 reflections
*c* = 11.9203 (8) Å	θ = 2.4–27.6°
α = 80.409 (3)°	µ = 8.16 mm^−^^1^
β = 80.952 (3)°	*T* = 296 K
γ = 81.730 (3)°	Plate, colourless
*V* = 1090.92 (13) Å^3^	0.32 × 0.20 × 0.20 mm

### Data collection

**Table:**

Bruker SMART CCD area-detector diffractometer	4994 independent reflections
Radiation source: fine-focus sealed tube	4689 reflections with *I* > 2σ(*I*)
Graphite monochromator	*R*_int_ = 0.046
phi and ω scans	θ_max_ = 27.6°, θ_min_ = 1.8°
Absorption correction: multi-scan (*SADABS*; Sheldrick, 1996)	*h* = −11→10
*T*_min_ = 0.180, *T*_max_ = 0.292	*k* = −13→8
8311 measured reflections	*l* = −15→12

### Refinement

**Table:**

Refinement on *F*^2^	Secondary atom site location: difference Fourier map
Least-squares matrix: full	Hydrogen site location: inferred from neighbouring sites
*R*[*F*^2^ > 2σ(*F*^2^)] = 0.040	H-atom parameters constrained
*wR*(*F*^2^) = 0.104	*w* = 1/[σ^2^(*F*_o_^2^) + (0.060*P*)^2^] where *P* = (*F*_o_^2^ + 2*F*_c_^2^)/3
*S* = 1.05	(Δ/σ)_max_ = 0.001
4994 reflections	Δρ_max_ = 3.29 e Å^−^^3^
335 parameters	Δρ_min_ = −4.30 e Å^−^^3^
0 restraints	Extinction correction: *SHELXL97* (Sheldrick, 2008), Fc^*^=kFc[1+0.001xFc^2^λ^3^/sin(2θ)]^-1/4^
Primary atom site location: structure-invariant direct methods	Extinction coefficient: 0.0028 (6)

### Special details

**Table:**

Geometry. All e.s.d.'s (except the e.s.d. in the dihedral angle between two l.s. planes) are estimated using the full covariance matrix. The cell e.s.d.'s are taken into account individually in the estimation of e.s.d.'s in distances, angles and torsion angles; correlations between e.s.d.'s in cell parameters are only used when they are defined by crystal symmetry. An approximate (isotropic) treatment of cell e.s.d.'s is used for estimating e.s.d.'s involving l.s. planes.
Refinement. Refinement of *F*^2^ against ALL reflections. The weighted *R*-factor *wR* and goodness of fit *S* are based on *F*^2^, conventional *R*-factors *R* are based on *F*, with *F* set to zero for negative *F*^2^. The threshold expression of *F*^2^ > σ(*F*^2^) is used only for calculating *R*-factors(gt) *etc*. and is not relevant to the choice of reflections for refinement. *R*-factors based on *F*^2^ are statistically about twice as large as those based on *F*, and *R*- factors based on ALL data will be even larger.

### Fractional atomic coordinates and isotropic or equivalent isotropic displacement parameters (Å^2^)

**Table:**

	*x*	*y*	*z*	*U*_iso_*/*U*_eq_	
Bi1	0.03927 (2)	0.317631 (15)	0.205035 (14)	0.00719 (10)	
O1	−0.2391 (5)	0.0131 (4)	0.3621 (3)	0.0160 (9)	
O2	−0.1181 (5)	0.1617 (4)	0.2430 (3)	0.0133 (8)	
O3	0.1632 (5)	0.4275 (4)	0.3440 (3)	0.0113 (8)	
O4	0.1835 (5)	0.4423 (4)	0.5264 (3)	0.0131 (8)	
O5	0.2417 (5)	0.1750 (4)	0.2585 (3)	0.0111 (8)	
O6	0.4205 (5)	0.0122 (4)	0.2244 (4)	0.0176 (9)	
O7	−0.1387 (6)	0.2967 (4)	−0.1314 (4)	0.0222 (10)	
O8	−0.1157 (5)	0.3531 (4)	0.0370 (3)	0.0119 (8)	
O9	−0.2156 (5)	0.4615 (4)	0.2792 (3)	0.0138 (8)	
H9B	−0.2893	0.4199	0.3131	0.017*	
H9A	−0.2206	0.5148	0.3251	0.017*	
O10	0.2651 (5)	0.4062 (4)	0.0543 (4)	0.0186 (9)	
H10A	0.3557	0.4188	0.0631	0.022*	
H10B	0.2214	0.4749	0.0209	0.022*	
O11	0.4567 (5)	0.6816 (4)	0.6148 (4)	0.0148 (9)	
N1	−0.0245 (5)	0.2448 (4)	0.4131 (4)	0.0075 (9)	
N2	0.1249 (6)	0.1743 (4)	0.0663 (4)	0.0097 (9)	
N3	0.3864 (6)	0.5920 (5)	0.3084 (4)	0.0142 (10)	
H3C	0.3117	0.5461	0.3175	0.017*	
N4	0.5959 (6)	0.8405 (5)	0.5319 (4)	0.0158 (10)	
H4A	0.6050	0.8601	0.5974	0.019*	
H4B	0.6371	0.8827	0.4697	0.019*	
C1	−0.1618 (6)	0.1040 (5)	0.3427 (4)	0.0089 (10)	
C2	−0.1153 (7)	0.1532 (5)	0.4435 (5)	0.0098 (10)	
C3	−0.1650 (7)	0.1078 (5)	0.5570 (4)	0.0114 (11)	
H3	−0.2275	0.0427	0.5763	0.014*	
C4	−0.1177 (7)	0.1637 (5)	0.6417 (5)	0.0109 (11)	
H4	−0.1519	0.1381	0.7189	0.013*	
C5	−0.0206 (7)	0.2567 (5)	0.6103 (4)	0.0104 (10)	
H5	0.0136	0.2930	0.6659	0.013*	
C6	0.0257 (6)	0.2955 (5)	0.4942 (4)	0.0079 (10)	
C7	0.1335 (7)	0.3969 (5)	0.4522 (5)	0.0094 (10)	
C8	−0.0775 (7)	0.2855 (5)	−0.0444 (5)	0.0133 (11)	
C9	0.0580 (7)	0.1811 (5)	−0.0284 (5)	0.0098 (11)	
C10	0.1121 (7)	0.0952 (5)	−0.1054 (4)	0.0108 (11)	
H10	0.0667	0.1011	−0.1719	0.013*	
C11	0.2330 (7)	0.0013 (5)	−0.0831 (5)	0.0135 (12)	
H11	0.2693	−0.0563	−0.1340	0.016*	
C12	0.3002 (7)	−0.0059 (5)	0.0177 (5)	0.0115 (11)	
H12	0.3796	−0.0693	0.0365	0.014*	
C13	0.2436 (6)	0.0850 (5)	0.0879 (4)	0.0094 (10)	
C14	0.3107 (7)	0.0891 (5)	0.1975 (5)	0.0109 (11)	
C32	0.5185 (7)	0.7455 (5)	0.5271 (5)	0.0125 (11)	
C34	0.5059 (7)	0.7129 (5)	0.4113 (5)	0.0107 (11)	
C35	0.4079 (8)	0.6240 (6)	0.4082 (5)	0.0147 (12)	
H35	0.3561	0.5859	0.4766	0.018*	
C37	0.4620 (7)	0.6414 (5)	0.2073 (5)	0.0136 (11)	
H37	0.4458	0.6170	0.1393	0.016*	
C38	0.5641 (7)	0.7287 (6)	0.2051 (5)	0.0150 (12)	
H38	0.6185	0.7618	0.1358	0.018*	
C39	0.5849 (7)	0.7670 (5)	0.3073 (5)	0.0129 (11)	
H39	0.6505	0.8277	0.3064	0.016*	
O1S	0.5518 (6)	0.3379 (5)	0.1336 (4)	0.0270 (11)	
H1B	0.6481	0.3356	0.1081	0.032*	
H1A	0.5374	0.3208	0.2064	0.032*	

### Atomic displacement parameters (Å^2^)

**Table:**

	*U*^11^	*U*^22^	*U*^33^	*U*^12^	*U*^13^	*U*^23^
Bi1	0.00731 (14)	0.00714 (14)	0.00735 (13)	−0.00166 (8)	−0.00141 (8)	−0.00063 (8)
O1	0.019 (2)	0.018 (2)	0.0128 (19)	−0.0104 (18)	−0.0017 (16)	−0.0039 (16)
O2	0.018 (2)	0.014 (2)	0.0088 (18)	−0.0081 (16)	0.0014 (15)	−0.0026 (15)
O3	0.012 (2)	0.0117 (18)	0.0110 (18)	−0.0058 (15)	−0.0016 (15)	−0.0009 (14)
O4	0.015 (2)	0.014 (2)	0.0138 (19)	−0.0046 (16)	−0.0030 (16)	−0.0070 (15)
O5	0.011 (2)	0.0128 (19)	0.0105 (18)	−0.0005 (15)	−0.0028 (15)	−0.0027 (14)
O6	0.016 (2)	0.019 (2)	0.017 (2)	0.0010 (18)	−0.0045 (17)	−0.0019 (17)
O7	0.022 (3)	0.028 (2)	0.017 (2)	0.0076 (19)	−0.0132 (18)	−0.0071 (18)
O8	0.012 (2)	0.0135 (19)	0.0103 (18)	0.0005 (16)	−0.0034 (15)	−0.0024 (14)
O9	0.013 (2)	0.014 (2)	0.0145 (19)	−0.0026 (16)	−0.0004 (16)	−0.0021 (15)
O10	0.013 (2)	0.018 (2)	0.022 (2)	−0.0065 (17)	−0.0003 (17)	0.0063 (16)
O11	0.013 (2)	0.015 (2)	0.016 (2)	−0.0055 (17)	0.0007 (16)	−0.0013 (16)
N1	0.008 (2)	0.007 (2)	0.008 (2)	−0.0020 (17)	−0.0009 (17)	−0.0017 (16)
N2	0.012 (2)	0.009 (2)	0.009 (2)	−0.0036 (18)	−0.0046 (18)	0.0014 (16)
N3	0.015 (3)	0.010 (2)	0.017 (2)	−0.0013 (19)	−0.003 (2)	0.0020 (18)
N4	0.020 (3)	0.017 (2)	0.012 (2)	−0.009 (2)	−0.0027 (19)	0.0001 (18)
C1	0.007 (3)	0.008 (2)	0.011 (2)	0.0050 (19)	−0.0028 (19)	−0.0028 (19)
C2	0.010 (3)	0.005 (2)	0.014 (3)	0.002 (2)	−0.003 (2)	−0.0034 (19)
C3	0.014 (3)	0.012 (3)	0.009 (2)	−0.005 (2)	−0.002 (2)	0.0010 (19)
C4	0.012 (3)	0.011 (3)	0.008 (2)	0.000 (2)	0.000 (2)	0.001 (2)
C5	0.011 (3)	0.010 (2)	0.010 (2)	−0.001 (2)	−0.003 (2)	0.0004 (19)
C6	0.005 (2)	0.007 (2)	0.010 (2)	0.0031 (19)	−0.0012 (19)	0.0011 (18)
C7	0.008 (3)	0.008 (2)	0.013 (3)	0.001 (2)	−0.003 (2)	−0.002 (2)
C8	0.009 (3)	0.016 (3)	0.015 (3)	−0.002 (2)	−0.004 (2)	−0.001 (2)
C9	0.011 (3)	0.009 (3)	0.009 (2)	−0.002 (2)	0.000 (2)	−0.0022 (19)
C10	0.012 (3)	0.013 (3)	0.009 (2)	−0.003 (2)	−0.001 (2)	−0.0032 (19)
C11	0.011 (3)	0.014 (3)	0.017 (3)	−0.005 (2)	0.003 (2)	−0.008 (2)
C12	0.008 (3)	0.011 (3)	0.016 (3)	−0.003 (2)	0.001 (2)	−0.001 (2)
C13	0.008 (3)	0.011 (2)	0.008 (2)	−0.005 (2)	0.0013 (19)	0.0006 (19)
C14	0.013 (3)	0.009 (3)	0.011 (2)	−0.003 (2)	−0.003 (2)	−0.0007 (19)
C32	0.009 (3)	0.012 (3)	0.015 (3)	0.001 (2)	−0.001 (2)	0.001 (2)
C34	0.008 (3)	0.008 (2)	0.015 (3)	0.003 (2)	−0.001 (2)	0.000 (2)
C35	0.016 (3)	0.013 (3)	0.014 (3)	−0.001 (2)	−0.002 (2)	0.002 (2)
C37	0.016 (3)	0.011 (3)	0.016 (3)	−0.001 (2)	−0.008 (2)	−0.003 (2)
C38	0.013 (3)	0.013 (3)	0.017 (3)	−0.004 (2)	0.002 (2)	0.001 (2)
C39	0.010 (3)	0.010 (3)	0.018 (3)	−0.002 (2)	−0.003 (2)	0.001 (2)
O1S	0.016 (2)	0.044 (3)	0.019 (2)	−0.009 (2)	−0.0021 (18)	0.006 (2)

### Geometric parameters (Å, º)

**Table:**

Bi1—O2	2.271 (4)	N4—H4B	0.8600
Bi1—O5	2.274 (4)	C1—C2	1.524 (7)
Bi1—N2	2.412 (4)	C2—C3	1.384 (7)
Bi1—N1	2.475 (4)	C3—C4	1.400 (7)
Bi1—O8	2.541 (4)	C3—H3	0.9300
Bi1—O10	2.630 (4)	C4—C5	1.376 (7)
Bi1—O3	2.631 (4)	C4—H4	0.9300
Bi1—O9	2.651 (4)	C5—C6	1.391 (7)
O1—C1	1.241 (6)	C5—H5	0.9300
O2—C1	1.276 (6)	C6—C7	1.520 (7)
O3—C7	1.272 (7)	C8—C9	1.528 (8)
O4—C7	1.242 (6)	C9—C10	1.396 (7)
O5—C14	1.297 (7)	C10—C11	1.383 (8)
O6—C14	1.223 (7)	C10—H10	0.9300
O7—C8	1.221 (7)	C11—C12	1.406 (8)
O8—C8	1.284 (7)	C11—H11	0.9300
O9—H9B	0.8500	C12—C13	1.383 (7)
O9—H9A	0.8499	C12—H12	0.9300
O10—H10A	0.8500	C13—C14	1.524 (7)
O10—H10B	0.8498	C32—C34	1.504 (8)
O11—C32	1.245 (7)	C34—C35	1.385 (8)
N1—C2	1.327 (6)	C34—C39	1.401 (8)
N1—C6	1.344 (6)	C35—H35	0.9300
N2—C13	1.339 (7)	C37—C38	1.385 (8)
N2—C9	1.339 (7)	C37—H37	0.9300
N3—C35	1.340 (8)	C38—C39	1.395 (8)
N3—C37	1.346 (8)	C38—H38	0.9300
N3—H3C	0.8600	C39—H39	0.9300
N4—C32	1.322 (7)	O1S—H1B	0.8500
N4—H4A	0.8600	O1S—H1A	0.8500
			
O2—Bi1—O5	90.01 (15)	C2—C3—C4	117.5 (5)
O2—Bi1—N2	71.89 (14)	C2—C3—H3	121.3
O5—Bi1—N2	68.92 (14)	C4—C3—H3	121.3
O2—Bi1—N1	67.23 (13)	C5—C4—C3	119.8 (5)
O5—Bi1—N1	73.07 (14)	C5—C4—H4	120.1
N2—Bi1—N1	122.99 (14)	C3—C4—H4	120.1
O2—Bi1—O8	74.88 (13)	C4—C5—C6	119.0 (5)
O5—Bi1—O8	134.11 (12)	C4—C5—H5	120.5
N2—Bi1—O8	65.21 (14)	C6—C5—H5	120.5
N1—Bi1—O8	133.40 (13)	N1—C6—C5	121.0 (5)
O2—Bi1—O10	142.16 (13)	N1—C6—C7	116.6 (4)
O5—Bi1—O10	80.74 (14)	C5—C6—C7	122.3 (5)
N2—Bi1—O10	70.54 (14)	O4—C7—O3	126.2 (5)
N1—Bi1—O10	140.96 (14)	O4—C7—C6	117.2 (5)
O8—Bi1—O10	85.52 (13)	O3—C7—C6	116.6 (5)
O2—Bi1—O3	130.76 (12)	O7—C8—O8	126.5 (6)
O5—Bi1—O3	75.54 (13)	O7—C8—C9	118.0 (5)
N2—Bi1—O3	137.93 (14)	O8—C8—C9	115.5 (5)
N1—Bi1—O3	63.53 (12)	N2—C9—C10	120.1 (5)
O8—Bi1—O3	145.34 (12)	N2—C9—C8	116.2 (5)
O10—Bi1—O3	82.39 (12)	C10—C9—C8	123.7 (5)
O2—Bi1—O9	84.07 (14)	C11—C10—C9	120.1 (5)
O5—Bi1—O9	145.08 (13)	C11—C10—H10	119.9
N2—Bi1—O9	139.30 (14)	C9—C10—H10	119.9
N1—Bi1—O9	72.92 (14)	C10—C11—C12	119.0 (5)
O8—Bi1—O9	77.23 (12)	C10—C11—H11	120.5
O10—Bi1—O9	123.14 (13)	C12—C11—H11	120.5
O3—Bi1—O9	82.50 (12)	C13—C12—C11	117.5 (6)
C1—O2—Bi1	125.2 (3)	C13—C12—H12	121.3
C7—O3—Bi1	120.2 (3)	C11—C12—H12	121.3
C14—O5—Bi1	123.2 (3)	N2—C13—C12	122.9 (5)
C8—O8—Bi1	120.5 (4)	N2—C13—C14	115.0 (5)
Bi1—O9—H9B	113.8	C12—C13—C14	122.1 (5)
Bi1—O9—H9A	125.5	O6—C14—O5	124.2 (5)
H9B—O9—H9A	99.6	O6—C14—C13	120.1 (5)
Bi1—O10—H10A	130.4	O5—C14—C13	115.8 (5)
Bi1—O10—H10B	103.5	O11—C32—N4	122.4 (5)
H10A—O10—H10B	107.7	O11—C32—C34	118.9 (5)
C2—N1—C6	119.9 (4)	N4—C32—C34	118.7 (5)
C2—N1—Bi1	117.0 (3)	C35—C34—C39	118.3 (5)
C6—N1—Bi1	123.1 (3)	C35—C34—C32	117.2 (5)
C13—N2—C9	120.3 (5)	C39—C34—C32	124.5 (5)
C13—N2—Bi1	117.1 (3)	N3—C35—C34	121.1 (5)
C9—N2—Bi1	122.6 (4)	N3—C35—H35	119.4
C35—N3—C37	121.8 (5)	C34—C35—H35	119.4
C35—N3—H3C	111.5	N3—C37—C38	119.7 (5)
C37—N3—H3C	126.1	N3—C37—H37	120.2
C32—N4—H4A	120.0	C38—C37—H37	120.2
C32—N4—H4B	120.0	C37—C38—C39	119.8 (5)
H4A—N4—H4B	120.0	C37—C38—H38	120.1
O1—C1—O2	124.8 (5)	C39—C38—H38	120.1
O1—C1—C2	119.0 (5)	C38—C39—C34	119.2 (5)
O2—C1—C2	116.2 (4)	C38—C39—H39	120.4
N1—C2—C3	122.7 (5)	C34—C39—H39	120.4
N1—C2—C1	114.2 (4)	H1B—O1S—H1A	111.1
C3—C2—C1	123.1 (5)		
			
O5—Bi1—O2—C1	68.1 (4)	C6—N1—C2—C1	−179.4 (5)
N2—Bi1—O2—C1	135.9 (5)	Bi1—N1—C2—C1	2.0 (6)
N1—Bi1—O2—C1	−3.5 (4)	O1—C1—C2—N1	175.8 (5)
O8—Bi1—O2—C1	−155.8 (5)	O2—C1—C2—N1	−4.8 (7)
O10—Bi1—O2—C1	142.9 (4)	O1—C1—C2—C3	−5.1 (8)
O3—Bi1—O2—C1	−2.7 (5)	O2—C1—C2—C3	174.3 (5)
O9—Bi1—O2—C1	−77.4 (4)	N1—C2—C3—C4	0.9 (9)
O2—Bi1—O3—C7	−2.2 (5)	C1—C2—C3—C4	−178.1 (5)
O5—Bi1—O3—C7	−79.4 (4)	C2—C3—C4—C5	−2.4 (9)
N2—Bi1—O3—C7	−112.2 (4)	C3—C4—C5—C6	1.6 (9)
N1—Bi1—O3—C7	−1.4 (4)	C2—N1—C6—C5	−2.4 (8)
O8—Bi1—O3—C7	127.6 (4)	Bi1—N1—C6—C5	176.1 (4)
O10—Bi1—O3—C7	−161.7 (4)	C2—N1—C6—C7	178.1 (5)
O9—Bi1—O3—C7	73.3 (4)	Bi1—N1—C6—C7	−3.4 (6)
O2—Bi1—O5—C14	71.8 (4)	C4—C5—C6—N1	0.9 (8)
N2—Bi1—O5—C14	1.3 (4)	C4—C5—C6—C7	−179.6 (5)
N1—Bi1—O5—C14	137.9 (4)	Bi1—O3—C7—O4	−179.4 (5)
O8—Bi1—O5—C14	3.1 (5)	Bi1—O3—C7—C6	0.3 (6)
O10—Bi1—O5—C14	−71.3 (4)	N1—C6—C7—O4	−178.4 (5)
O3—Bi1—O5—C14	−155.8 (4)	C5—C6—C7—O4	2.1 (8)
O9—Bi1—O5—C14	151.4 (4)	N1—C6—C7—O3	1.9 (7)
O2—Bi1—O8—C8	−79.2 (4)	C5—C6—C7—O3	−177.6 (5)
O5—Bi1—O8—C8	−4.3 (5)	Bi1—O8—C8—O7	−176.9 (5)
N2—Bi1—O8—C8	−2.5 (4)	Bi1—O8—C8—C9	3.2 (6)
N1—Bi1—O8—C8	−115.3 (4)	C13—N2—C9—C10	−0.2 (8)
O10—Bi1—O8—C8	68.2 (4)	Bi1—N2—C9—C10	−179.7 (4)
O3—Bi1—O8—C8	137.9 (4)	C13—N2—C9—C8	179.4 (5)
O9—Bi1—O8—C8	−166.4 (4)	Bi1—N2—C9—C8	−0.1 (6)
O2—Bi1—N1—C2	0.4 (4)	O7—C8—C9—N2	178.1 (5)
O5—Bi1—N1—C2	−97.0 (4)	O8—C8—C9—N2	−2.1 (7)
N2—Bi1—N1—C2	−47.2 (4)	O7—C8—C9—C10	−2.4 (8)
O8—Bi1—N1—C2	38.5 (5)	O8—C8—C9—C10	177.5 (5)
O10—Bi1—N1—C2	−147.0 (4)	N2—C9—C10—C11	1.2 (8)
O3—Bi1—N1—C2	−178.9 (4)	C8—C9—C10—C11	−178.3 (5)
O9—Bi1—N1—C2	91.0 (4)	C9—C10—C11—C12	−0.1 (8)
O2—Bi1—N1—C6	−178.2 (5)	C10—C11—C12—C13	−1.9 (8)
O5—Bi1—N1—C6	84.5 (4)	C9—N2—C13—C12	−2.0 (8)
N2—Bi1—N1—C6	134.2 (4)	Bi1—N2—C13—C12	177.5 (4)
O8—Bi1—N1—C6	−140.0 (4)	C9—N2—C13—C14	179.1 (4)
O10—Bi1—N1—C6	34.4 (5)	Bi1—N2—C13—C14	−1.3 (6)
O3—Bi1—N1—C6	2.5 (4)	C11—C12—C13—N2	3.1 (8)
O9—Bi1—N1—C6	−87.5 (4)	C11—C12—C13—C14	−178.2 (5)
O2—Bi1—N2—C13	−97.1 (4)	Bi1—O5—C14—O6	179.4 (4)
O5—Bi1—N2—C13	0.2 (3)	Bi1—O5—C14—C13	−2.4 (6)
N1—Bi1—N2—C13	−51.3 (4)	N2—C13—C14—O6	−179.3 (5)
O8—Bi1—N2—C13	−178.4 (4)	C12—C13—C14—O6	1.8 (8)
O10—Bi1—N2—C13	87.5 (4)	N2—C13—C14—O5	2.4 (7)
O3—Bi1—N2—C13	34.5 (5)	C12—C13—C14—O5	−176.5 (5)
O9—Bi1—N2—C13	−153.9 (3)	O11—C32—C34—C35	8.6 (8)
O2—Bi1—N2—C9	82.5 (4)	N4—C32—C34—C35	−172.0 (6)
O5—Bi1—N2—C9	179.7 (4)	O11—C32—C34—C39	−172.1 (6)
N1—Bi1—N2—C9	128.2 (4)	N4—C32—C34—C39	7.3 (9)
O8—Bi1—N2—C9	1.2 (4)	C37—N3—C35—C34	2.0 (9)
O10—Bi1—N2—C9	−92.9 (4)	C39—C34—C35—N3	−1.3 (9)
O3—Bi1—N2—C9	−146.0 (4)	C32—C34—C35—N3	178.1 (5)
O9—Bi1—N2—C9	25.7 (5)	C35—N3—C37—C38	−0.6 (9)
Bi1—O2—C1—O1	−174.8 (4)	N3—C37—C38—C39	−1.5 (9)
Bi1—O2—C1—C2	5.8 (7)	C37—C38—C39—C34	2.2 (9)
C6—N1—C2—C3	1.5 (8)	C35—C34—C39—C38	−0.8 (9)
Bi1—N1—C2—C3	−177.1 (4)	C32—C34—C39—C38	179.9 (6)

### Hydrogen-bond geometry (Å, º)

Cg1 is the centroid of the N2/C9–C13 ring.

**Table:**

*D*—H···*A*	*D*—H	H···*A*	*D*···*A*	*D*—H···*A*
O1*S*—H1*A*···O11^i^	0.85	2.14	2.961 (7)	164
O1*S*—H1*B*···O8^ii^	0.85	2.13	2.979 (7)	173
N3—H3*C*···O3	0.86	1.91	2.766 (7)	173
N4—H4*A*···O5^i^	0.86	2.29	3.035 (6)	144
N4—H4*B*···O1^iii^	0.86	2.05	2.874 (6)	160
O9—H9*A*···O4^iv^	0.85	1.99	2.760 (5)	151
O9—H9*B*···O11^iv^	0.85	1.96	2.811 (6)	177
O10—H10*A*···O1*S*	0.85	2.05	2.782 (7)	144
O10—H10*B*···O8^v^	0.85	2.02	2.860 (6)	172
C5—H5···O7^vi^	0.93	2.57	3.170 (7)	122
C11—H11···O2^vii^	0.93	2.49	3.159 (7)	129
C35—H35···O4	0.93	2.27	3.004 (8)	136
C39—H39···O1^iii^	0.93	2.57	3.456 (7)	160
C38—H38···*Cg*1^viii^	0.93	2.70	3.549 (7)	153

Symmetry codes: (i) −*x*+1, −*y*+1, −*z*+1; (ii) *x*+1, *y*, *z*; (iii) *x*+1, *y*+1, *z*; (iv) −*x*, −*y*+1, −*z*+1; (v) −*x*, −*y*+1, −*z*; (vi) *x*, *y*, *z*+1; (vii) −*x*, −*y*, −*z*; (viii) −*x*+1, −*y*+1, −*z*.
